# Trap Assisted Dynamic Mechanoluminescence Toward Self‐Referencing and Visualized Strain Sensing

**DOI:** 10.1002/advs.202410673

**Published:** 2024-11-27

**Authors:** Tianli Wang, Pengfei Zhang, Jianqiang Xiao, Ziyi Guo, Xiongwu Xie, Jiahao Huang, Jiaojiao Zheng, Xuhui Xu, Lei Zhao

**Affiliations:** ^1^ School of Physics and Opto‐Electronic Technology Collaborative Innovation Center of Rare‐Earth Optical Functional Materials and Devices Development Baoji University of Arts and Sciences Baoji Shaanxi 721016 P. R. China; ^2^ College of Materials Science and Engineering Key Laboratory of Advanced Materials of Yunnan Province Kunming University of Science and Technology Kunming Yunnan 650093 P. R. China

**Keywords:** dynamic, mechanoluminescence, self‐referencing, strain sensing, visualizion

## Abstract

Strain sensors utilizing mechanoluminescent (ML) materials have garnered significant attention and application due to their advantages, such as self‐powering, non‐contact operation, and real‐time response. However, ML‐based strain sensing techniques typically rely on the establishing of a mathematical relationship between ML intensity and mechanical parameters. The absolute ML intensity is vulnerable to environmental factors, which can result in measurement errors. Herein, an color‐resolved visualized dynamic ML and self‐referencing strain sensing is investigated in Ca_9_Al(PO_4_)_7_: Tb^3+^, Mn^2+^. By analyzing the ML performance under various mechanical stimulations and adjustable strain parameters, a relationship between strain and the ML intensity ratio of Tb^3+^/Mn^2+^ is aimed to bed established. This will enable the development of a self‐referencing and visualized strain sensing technology. Through a comparison of luminescence characteristics under continuous mechanical stimulation (stretching) and continuous X‐ray irradiation, it is discovered that the ratiometric dynamic ML is primarily driven by the dynamic filling and continuous release of carriers form traps, which compensates for the ML of Mn^2+^. Leveraging the self‐referencing and color‐resolved (from green to red) visualized ML characteristics, an application scenario for monitoring human joint movement is developed. This approach offers new insights into the use of dynamic ML materials in strain sensing and human‐machine interaction.

## Graphical abstract

In this work, an color‐resolved visualized dynamic ML and self‐referencing strain sensing is investigated in Ca_9_Al(PO_4_)_7_: Tb^3+^, Mn^2+^. The ratiometric ML is primarily driven by the trapped carriers compensates for the ML of Mn^2+^. Finally, real‐time monitoring of human joint quantification is achieved through human‐machine interaction, providing new ideas and methods for the strain sening.

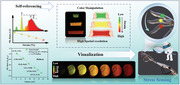

## 1 Introduction

With the development of technology, the usage and application scenarios of strain sensors have significantly increased, leading to more stringent requirements for these sensors.^[^ ^1^, ^2^, ^3^ ^]^ Traditional resistive and capacitive strain sensors rely on electronic signals, making them susceptible to electromagnetic interference, complex wiring, stray capacitance, and thermal disturbances.^[^ ^4^ ^]^ In recent years, ML materials, due to their self‐charging, non‐contact, and visual characteristics, have emerged as a promising solution to overcome these issues. ML materials also offer higher spatial resolution, making them the subject of extensive research.^[^ ^5^, ^6^, ^7^, ^8^ ^]^

Currently, the prevalent approach involves establishing a correlation between the absolute intensity of ML and stress/strain, enabling the development of ML‐based mechanical sensing technology.^[^ ^9^ ^]^ However, optical sensing techniques based on the absolute intensity of ML have limitations, they are susceptible to interference from environmental factors such as moisture, temperature, acidity, and alkalinity. They also suffer from issues of insufficient accuracy, large errors, limited reliability, and difficulties in visualization. In 2021, Wang et al. achieved stress/strain‐induced ML color control in the Sr_2_P_2_O_7_: Eu,Y by introducing two different luminescent centers, Eu^2+^/Eu^3+^. They successfully realized semi‐quantitative visual mechanical sensing by leveraging the high reliability of ML color.^[^ ^10^ ^]^ To overcome the instability of absolute ML intensity, the ratiometric ML has gradually been proposed. Cheng et al. proposed a new strategy for ratiometric ML by stress‐induced change in local site symmetry and dual‐activator mode.^[^ ^9^, ^11^ ^]^ These works provide a novel approach for the design and application of self‐referencing ratiometric ML.^[^ ^12^, ^13^, ^14^, ^15^, ^16^ ^]^

Strain is generated during the continuous application of force. The ability to produce dynamic ratiometric ML under sustained force or continuous excitation could enable self‐referencing strain sensing.^[^ ^17^ ^]^ Zhang et al. developed a series of novel materials with dynamic photoluminescence (PL) properties such as MgGa_2_O_4_,^[^ ^18^ ^]^ Na_2_CaGe_2_O_6_: Tb^3+[^ ^19^ ^]^ and CaZnGe_2_O_6_: Mn^2+^.^[^ ^20^ ^]^ During the continuous irradiation process, the color of PL was dynamical change can be generated due to the different trap states of various luminescent centers. For triboelectric ML, the process of tensile strain changes implies the continuous excitation of the triboelectric field. Under this sustained electric field excitation, generating dynamic ML with the aid of traps seems to be a feasible strategy.^[^ ^21^, ^22^, ^23^, ^24^ ^]^

In this study, we propose a novel strategy of utilizing lanthanide/transition metal activator combinations to achieve color‐resolved self‐referencing strain sensing. Ca_9_Al(PO_4_)_7_: Tb^3+^, Mn^2+^ and polydimethylsiloxane (PDMS) composite ML devices are used as the research subjects. By studying the ML sensitivity, self‐charging persistent ML (PersML) and traps state level under mechanical stimuli, the reliability mechanism of ML color changes is demonstrated. Furthermore, we achieve a self‐powered, self‐recoverable, and highly sensitive intensity ratio mechanical sensing technology based on human‐machine interaction response.

## 2 Results and Discussion

**Figure 1:**
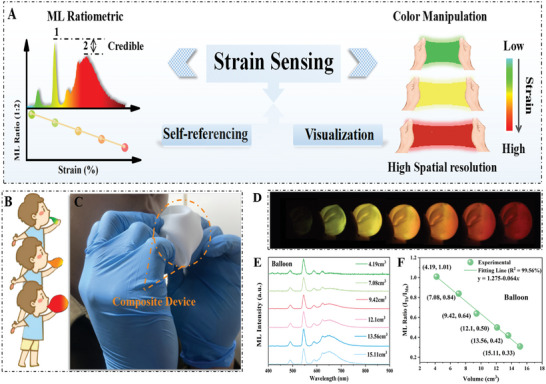
(A) Schematic diagram of visualized self‐referencing strain sensing based on dynamic ML. (B) Diagram of the balloon inflated by the composite device. (C) Schematic diagram of an experimental device for inflating a balloon fabricated by CAP: Tb^3+^, Mn^2+^@PDMS composite devices. (D) ML photos, (E) ML spectra, (F) ML integral intensity ratio (I_Tb_/I_Mn_) from the balloon at different gas volumes.

The design concept of the self‐referencing and visualized strain sensing material proposed in this work is illustrated in **Figure** **1**A. As previously mentioned, self‐referencing sensing technology based on ML intensity ratios offers greater reliability compared to absolute intensity measurements. Moreover, employing luminescent centers with significantly different emitting colors not only facilitates intensity ratio‐based sensing but also has the potential to achieve high spatial resolution in color‐resolved visualizations.^[^ ^25^ ^]^ In this work, we selected Tb^3+^ and Mn^2+^ ions as the luminescent centers, whereas the strain increases, the emission color of the sensor device changes from green to red. During this transition, the ML ratio between the two luminescent centers is not affected by the external environment. Additionally, the color change resulting from the variation in luminescent intensity ratio is more suitable for practical strain visualized sensing. To validate this concept, we conducted an experiment using a balloon fabricated by the CAP: Tb^3+^, Mn^2+^@ PDMS device, as shown in Figure 1B–D. During the experiment, we observed the deformation of the device as the balloon filled. This deformation caused the ML color transfered from green to orange‐yellow and then to red. Combining the experimental results with spectral data, Figure 1E,F display the ML spectra of balloons with different volumes and the corresponding ML integrated intensity ratios (I_Tb_/I_Mn_). The results indicate that the ML intensity of the sample increases with increasing applied strain, particularly the radiative transition of Mn^2+^ from ^4^A_1_→^6^T_1_ increases more rapidly. This can be observed from the relationship between the ML integrated intensity ratio (I_Tb_/I_Mn_) and balloon volume shown in Figure 1F, which decreases linearly with increasing applied strain. The linear fitting factor reaches 99.56%, providing sufficient evidence for the reliable stability of the intensity ratio sensing technology.

The qualitative and quantitative characteristics of the device are dependent on the structure of the ML phosphors and the inorganic‐organic polymer.^[^ ^21^ ^]^ XRD patterns of single‐doped and co‐doped phosphors with different doping concentrations are shown in Figures S1–S3 (Supporting Information). All samples exhibit diffraction peaks consistent with the standard data of Ca_9_Al(PO_4_)_7_ (JCPDS card No.48‐1192), without any impurity peaks. Specifically, Rietveld refinement was performed on the co‐doped sample of CAP: 0.4Tb^3+^, 0.03Mn^2+^, and the low R‐value further confirms that the doping of rare‐earth Tb^3+^ and transition metal Mn^2+^ ions did not alter the matrix structure, as shown in Figure S4 and Table S1 (Supporting Information). SEM‐EDS spectroscopic analysis revealed the uniform distribution of Ca, Al, P, O, Tb, and Mn elements, thereby confirming the successful incorporation of Tb^3+^ and Mn^2+^ ions into the matrix structure, as shown in Figure S5 (Supporting Information). The multi‐color luminescence of the device is determined by the choice of concentrations of rare‐earth and transition metal ions. Fluorescence spectroscopy analysis determined that the CAP: Tb^3+^, Mn^2+^ samples exhibited a dynamic luminescent intensity ratio I_Tb_/I_Mn_ (as shown in Figures S6–S13, Supporting Information).

The construction of the inorganic‐organic CAP: 0.4Tb^3+^, 0.03Mn^2+^@PDMS composite device involves magnetic fusion at room temperature followed by high‐temperature crosslinking, as depicted in Figure S14 (Supporting Information). X‐ray diffraction analysis of the device confirms the presence of diffraction peaks corresponding to both the organic polymer PDMS and the inorganic phosphor (Figure S15, Supporting Information). When combined with the prepared cross‐sectional images of the device, it is evident that the CAP: 0.4Tb^3+^, 0.03Mn^2+^@PDMS device exhibits uniform mixing, supporting subsequent qualitative and quantitative characterization experiments. The working of its involves withstanding different levels of force and vibration. Therefore, the mechanical properties, such as toughness and flexibility, are crucial for the sensor device.^[^ ^26^, ^27^ ^]^ The high flexibility and resilience of the device can be observed from the device photos showing two‐fold stretching and bending in Figure S16 (Supporting Information). Fitting calculations based on Hooke's law on the stress–strain curve yield an elasticity of 0.0073 for the composite elastomer. Thus, this highly flexible composite elastomer device holds great potential for strain sensing applications.

**Figure 2:**
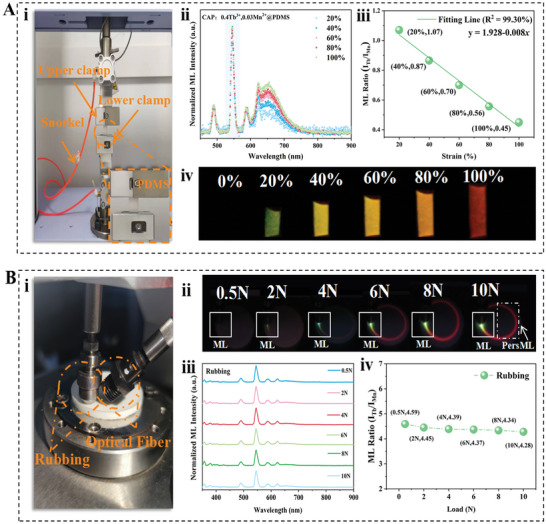
(A) (i) Schematic diagram of tensile device. (ii) Normalized ML spectra, (iii) ML integral intensity ratio (I_Tb_/I_Mn_), (iv) ML photo of CAP:0.4Tb^3+^, 0.03Mn^2+^@PDMS composite device under varying strain, respectively. (B) (i) Schematic diagram of friction device. (ii) Friction ML photo, (iii) Friction ML spectra, (iv) Friction ML integral intensity ratio (I_Tb_/I_Mn_) of CAP:0.4Tb^3+^, 0.03Mn^2+^@ PDMS composite devices under different friction loads, respectively.

There are various methods for testing ML. To qualitatively distinguish the effects of different mechanical stimuli on the ML properties, we conducted both tensile and friction tests on the composite devices. **Figure** **2**A (i) illustrates the schematic of the tensile device. The normalized ML spectra under different strains reveal that the red emission peak intensity at 648 nm dynamically increases with increasing strain of the CAP: 0.4Tb^3+^, 0.03Mn^2+^@PDMS composite device (Figure 2A (ii)). Consequently, the ML color of the sample exhibits dynamic changes, transferring from green to yellow, orange, and finally to red (Figure 2A (iv)). The data results of different strain are represented by the ML intensity ratio I_Tb_/I_Mn_, where I_Tb_ and I_Mn_ correspond to the integrated intensities of the Tb^3+ 5^D_4_→^7^F_5_ transition and the Mn^2+ 4^A_1_→^6^T_1_ transition, respectively.^[^ ^28^, ^29^ ^]^ The ML ratio for CAP: 0.4Tb^3+^, 0.03Mn^2+^@PDMS decreases from 1.07 (100%) to 0.45 (20%) as calculated from the ML intensity ratio of I_Tb_/I_Mn_. A linear fit based on the I_Tb_/I_Mn_ ML intensity ratio is given by the equation y = 1.928–0.008*x*, where *x* represents the strain and y represents the ML intensity ratio of I_Tb_/I_Mn_. The high fitting factor of 99.30% demonstrates the high sensitivity and precision of the sensor.

Figure 2B. (i) presents the friction test device. The friction setup consists of a compression friction pair and a rotating disc, and the emitted light from the sensor device is collected through a fiber optic spectrometer. The ML photos captured during friction under different forces for the composite elastomer are shown in Figure 2B (ii). Upon visual observation, under different friction loads, the ML intensity changes significantly, while the ML color remains nearly unchanged. The corresponding ML spectra for different friction forces are shown in Figure 2B (iii), they are almost entirely characterized by the typical emission peaks of Tb^3+^. We surmise that this is due to the discontinuous nature of the friction experiment where the force application is not continuous and the location of force is constantly changing. In contrast, the tensile experiment involves continuous strain application to the same device, compared to the transient nature of friction, the process of stretching involves continuous excitation. Figure 2B (iv) displays the ML integrated intensity ratio for different applied loads. The ML integrated intensity ratios for CAP: 0.4Tb^3+^, 0.03Mn^2+^@PDMS decreases from 4.59 (10N) to 4.28 (0.5N), indicating a minor change in the ML ratio. Notably, during the friction process, we observed that the red persistent ML (PersML) becomes stronger with increasing force (dashed box). PersML has infinite potential for applications in motion detection and delay detection, and this phenomenon is closely related to the color‐resolved mechanism of the device, which will be discussed later. These results demonstrate that the ML characteristics vary significantly under different mechanical stimulation, which suggests potential applications for this device in qualitatively distinguishing between different types of mechanical stimulation.

**Figure 3:**
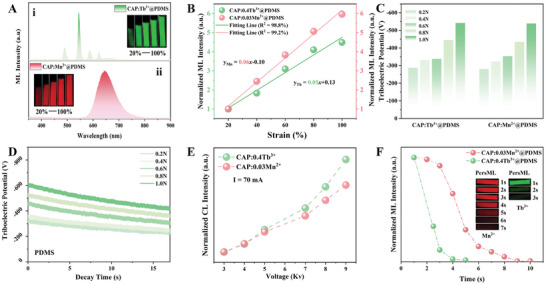
(A) ML spectra of (i) CAP: Tb^3+^ @PDMS, (ii) CAP: Mn^2+^@PDMS, the illustrations are ML photos taken under different strain. (B) The relationship between the ML intensity and the strain of CAP: Tb^3+^@ PDMS and CAP: Mn^2+^ @PDMS. (C) Frictional potentials under different load of CAP: Tb^3+^ @PDMS and CAP: Mn^2+^ @PDMS. (D) Time‐dependent frictional potential under different load. (E) The relationship between CL intensity and excitation voltage of CAP: Tb^3+^ and CAP: Mn^2+^. (F) Time‐dependent PersML intensity of CAP: Tb^3+^ @PDMS and CAP: Mn^2+^ @PDMS, the illustration is the corresponding photos.

In order to gain a deeper understanding the mechanism the ML phenomenon of CAP: Tb^3+^, Mn^2+^@PDMS under different force stimulation modes, we first investigated the ML phenomenon and mechanism of single‐doped CAP: Tb^3+^@PDMS and CAP: Mn^2+^@PDMS. Figure S17 (Supporting Information) shows the ML photos of CAP: 0.4Tb^3+^@PDMS and CAP: 0.03Mn^2+^@PDMS under different mechanical stimuli (compression, tensile, and tearing), demonstrating that these materials are highly sensitive to various mechanical behavior, with intense green and red emissions. The corresponding ML spectra in **Figure** **3**A (i,ii) confirm that the ML of CAP: Tb^3+^@PDMS and CAP: Mn^2+^@PDMS originates from the same luminescent center as the PL (Figures S7 and S9, Supporting Information), with intense green emission corresponding to Tb^3+^ (^5^D_4_→^7^F_j_ (j = 6,5,4,3)) and red emission corresponding to Mn^2+^ (^4^T_1_→^6^A_1_) characteristic radiative transitions. The relationship between ML intensity and strain of CAP: Tb^3+^@PDMS and CAP: Mn^2+^@PDMS (Figure 3B) and the corresponding ML photos reveal that the ML intensity increased linearly with increasing strain, with fitting factors of 98.8% and 99.2%, respectively. Furthermore, for flexible ML devices, the elasticity and toughness of the material can be compromised by repeated mechanical stimuli due to the organic–inorganic material composite, leading to poorer ML performance and affecting the sensing accuracy. Therefore, the self‐recovery performance of the sensor device is crucial.^[^ ^30^ ^]^ Figures S18 and S19 (Supporting Information) demonstrates the self‐recovery performance of the CAP: 0.4Tb^3+^@PDMS device after being stretched to 100% deformation, with the ML intensity recovering to 93.06% of the initial intensity after waiting for 24 h and continuing the stretching under the same experimental conditions. After another cycle, the ML intensity can recover to 85.54% of the initial intensity. Similarly, for the CAP: Mn^2+^@PDMS device, the ML intensity recovers to 92.01% of the initial intensity after the first recovery and to 83.10% after the second recovery, demonstrating the excellent self‐recovery performance of the sensor device. In related studies conducted by the laboratory research group, it has been proven that the self‐recovery performance becomes even more excellent with increased recovery time and high‐temperature recovery.

We speculate that the triboelectric electricity induced by inorganic‐organic interfacial friction plays a significant role in the ML of CAP: 0.4Tb^3+^, 0.03Mn^2+^@PDMS.^[^ ^18^ ^]^ To further confirm this, we investigated the triboelectric potential of CAP: Tb^3+^@PDMS and CAP: 0Mn^2+^@PDMS under different friction loads, as shown in Figure 3C. The composite device is capable of generating charged electrons under friction, which are then transferred to the luminescent centers and converted into ML emission. The intensities of both the triboelectric potential difference and ML emission increases with higher friction forces, as shown in Figure 3D. In order to further explore the influence of triboelectric field induced ML on strain and strain rate, we measured the triboelectric potential across different speeds, as illustrated in Figure S20a (Supporting Information). Our results indicate that higher strain rates correspond to increased triboelectric potential. Similarly, we assessed the ML intensity under varying strain rates, also shown in Figure S20b (Supporting Information). The trend in ML intensity mirrored that of the triboelectric potential, with both increasing as strain rate heightened. This indicates that greater strain rates lead to higher triboelectric luminescence intensity, further demonstrating the substantial impact of strain rate on the absolute intensity of ML. We further tested the ML spectra and images of the composite device at different strain rates under 100% deformation, as shown in Figures S21 and S22 (Supporting Information). Our observations revealed that when the strain rate is low, the intensity of ML is weak, making the color change less perceptible to the naked eye. However, as the strain rate increases, both the ML intensity and the visibility of the color change improve, facilitating signal detection and photographic capture. In summary, the strain rate significantly affects the absolute strength of ML, this is logical since a higher deformation rate generates more friction charges over a given duration, resulting in increased ML intensity. Nonetheless, at varying strain rates, the material continues to exhibit color change, indicating trap‐assisted dynamic ML. This emission process is analogous to cathodoluminescence (CL), as depicted in Figures S23 and S24 (Supporting Information). All samples exhibit CL with the same luminescent centers as the PL and ML emissions, and the CL intensity correspondingly increases with the driving voltage, indicating the presence of an effective radiative pathway under high‐energy electron bombardment, as shown in Figure 3E. As previously mentioned, the CAP: Tb^3+^, Mn^2+^@PDMS device exhibits a phenomenon known as PersML.^[^ ^31^ ^]^ Therefore, we also studied the PersML performance of CAP: Tb^3+^@PDMS and CAP: Mn^2+^@PDMS (Figures S25 and S26, Supporting Information). By examining the PersML photos at different delay times in Figure 3F, we observed that the PersML duration of CAP: 0.03Mn^2+^@PDMS exceeds 7 s, whereas CAP: 0.4Tb^3+^@PDMS lasts less than 3 s. Additionally, the PersML intensity of CAP: Mn^2+^@PDMS is several orders of magnitude higher than that of CAP: Tb^3+^@PDMS, providing an explanation for the red PersML exhibited by the CAP: Tb^3+^, Mn^2+^@PDMS sensor device under friction.^[^ ^28^, ^32^ ^]^

**Figure 4:**
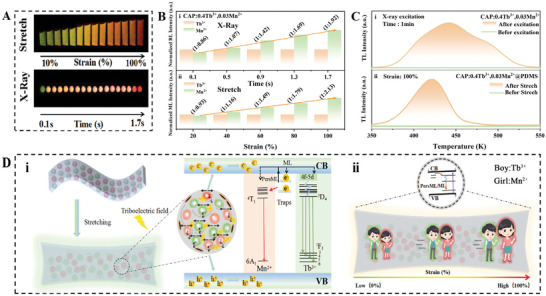
(A) The ML/RL photos, (B)(i), (ii) ML/RL intensity ratio (I_Tb_/I_Mn_) under various strain (10%100%) and different irradiated time by X‐ray of CAP: 0.4Tb^3+^, 0.03Mn^2+^@PDMS. (C) (i) TL spectra before and after stretching of CAP:0.4Tb^3+^, 0.03Mn^2+^@ PDMS. (ii) TL spectra at 1 min X‐ray pre‐irradiation and unexcited of CAP:0.4Tb^3+^, 0.03Mn^2+^. (D) (i) Mechanism diagram of ML and PersML. (ii) Schematic diagram of trap assisted dynamic ML.

To further elucidate the color‐resolved dynamic ML mechanism in CAP: Tb^3+^, Mn^2+^@PDMS devices, we conducted a comparative analysis of the results from tensile testing and continuous X‐ray irradiation, the ML and RL photos are shown in **Figure** **4**A. Through comparison, we observed that the RL generated under continuous X‐ray irradiation exhibited a color change phenomenon similar to that of the ML produced during tensile testing. By combining the ML/RL intensity ratio (I_Tb_/I_Mn_) in Figure 4B and the corresponding color evolution CIE coordinates (Figures S27 and S28 and Tables S2 and S3, Supporting Information), it becomes more visually apparent that the emission color of the sensor device shifts from green to red, representing a similar emission process as ML and X‐ray luminescence. PersML is predominantly caused by trap filling, so we measured the TL spectra of CAP: Tb^3+^, Mn^2+^ before and after stretching stimulation as shown in Figure 4C (ii). The sample did not show any TL signals before stretching, but after stretching stimulation, strong TL peaks at 350–450 K appeared, explaining the PersML phenomenon observed after strain unload. The TL spectra of CAP: 0.4Tb^3+^, 0.03Mn^2+^@PDMS before and after X‐ray excitation for 1 minute are shown in Figure 4C (i). The results are consistent with those from the tensile test. It is worth noting that X‐ray irradiation increases the number of traps and broadens the TL band. Overall, these phenomena further support the notion of the same emission process occurring during stretching and X‐ray excitation.^[^ ^33^, ^34^ ^]^ Finally, we propose a possible ML and PersML mechanism as illustrated schematically in Figure 4D (i). Where the relative displacement between the powder and PDMS generates a triboelectric field. The triboelectric field induces the separation and recombination of electron‐hole pairs, resulting in the emission of Tb^3+^ and Mn^2+^ ions as they transition from the valence band to the conduction band. This radiation transition gives rise to the ML phenomenon. Additionally, some electrons are transferred to trap levels and stored. Under ambient temperature thermal activation conditions, most of the electrons in shallow traps transfers to the excited states of Mn^2+^ ions, with a small fraction transfers to the excited states of Tb^3+^ ions, leading to the generation of PersML. The mechanism of ML differs from that of ZnS:Cu. Although ZnS possesses a non‐centrosymmetric crystal structure, the ML phenomenon observed after its combination with PDMS is influenced not only by the piezoelectric effect but also by the triboelectric effect.^[^ ^35^, ^36^, ^37^ ^]^ The non‐centrosymmetric nature of zinc sulfide generates a piezoelectric potential, particularly near the Cu ion sites, where the local piezoelectric field is likely stronger. This potential tilts the conduction band (CB) and valence band (VB) of zinc sulfide, enabling trapped electrons to excite into the CB. These trapped electrons subsequently recombine with holes through non‐radiative energy transfer. Concurrently, the released energy can be transferred to Cu ions, leading to their excitation and resulting in ML. Moreover, during the strain process, the relative displacement between ZnS:Cu particles and PDMS generates friction, which may facilitate the transfer of electrons from one surface to another, causing a local charge imbalance. The accumulated charge creates an electric field that influences the energy levels and movement of electrons within the material. When electrons return to the ground state under the influence of this electric field, energy is released in the form of light, producing triboelectroluminescence. Consequently, this mechanism contributes to a gradual enhancement of ML. Therefore, a schematic diagram illustrating the trap‐assisted dynamic ML of Tb^3+^‐Mn^2+^ ions in CAP: 0.4Tb^3+^, 0.03Mn^2+^@PDMS sensor devices are summarized (Figure 4D (ii)), where Tb^3+^ is represented by a boy and Mn^2+^ by a girl. During the stretching process from 0% to 100% strain, both Tb^3+^ and Mn^2+^ exhibit simultaneous increases in ML. The key difference is that Mn^2+^ can more effectively capture carriers within traps. During continuous stretching, the carriers released from these traps provide effective compensation for Mn^2+^ ML, which dynamically enhances its intensity more rapidly and ultimately leading to a color‐changing ML. This process is similar to the dynamic photoluminescence mentioned in the introduction.^[^ ^20^ ^]^

**Figure 5:**
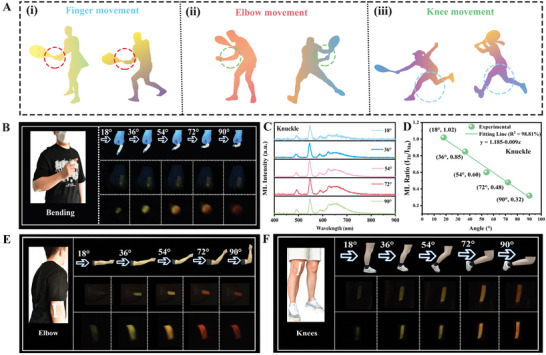
(A) (i‐iii): Diagram of different joint movements of the human body. (B) ML photos, (C)ML spectra, (D) ML integral intensity ratio (I_Tb_/I_Mn_) of CAP: Tb^3+^, Mn^2+^@ PDMS composite device in finger joint bending at different angles. (E) ML photos of CAP: Tb^3+^, Mn^2+^@PDMS composite device in the elbow and (F) knee joints bending at different angles.

In addition to the intensity ratio sensing technology under stretching stimulation, we also presented optical photos of CAP: Tb^3+^, Mn^2+^@PDMS under compression stimulation, as shown in Figure S29 (Supporting Information), which also exhibited a color change from green to red. Due to its excellent self‐referencing sensing performance under different force stimuli, this material can serve as a self‐powered physiological sensor.^[^ ^38^ ^]^ We successfully fabricated CAP: Tb^3+^, Mn^2+^@PDMS sensor devices and attached them to the wrists, fingers, and knees of volunteers, as shown in **Figure** **5**A. By observing the color change and the ratio of ML integration intensity, we can determine the activity status of human joints. Figure 5B illustrates the ML images of the finger joint at different bending angles under bright field, semi‐dark field, and dark field conditions. As the bending angle increases, the color of the composite elastomer changes from green to yellow and then to red, demonstrating good color resolution and sensitivity.^[^ ^39^, ^40^ ^]^ Figure 5C,D present the ML spectra and the corresponding ML integration intensity ratio (I_Tb_/I_Mn_) measured at different bending angles of the composite elastomer at the joint. The results indicate that the ML intensity increases as the bending angle increases. At an angle of 18°, the ML integration intensity ratio (I_Tb_/I_Mn_) of the sample was 1.02, while it is 0.32 at 90°, representing an increase in Mn^2+^ emission and a color shift toward red. The range of values within the 18–90° interval was reduced threefold, further demonstrating its high color resolution and sensitivity. Additionally, under bright field, semi‐dark field, and dark field conditions, we obtained ML images of the composite elastomer bent at different angles at the elbow joint (as shown in Figure 5E). Figures S30–S33 (Supporting Information) respectively display the ML spectra and the corresponding ML integration intensity ratio (I_Tb_/I_Mn_) measured at different bending angles of the sensor device at the elbow joint. The ML integration intensity ratio at the elbow joint ranges from 1.20 (angle: 18°) to 0.32 (angle: 90°), with a linear fitting factor of 98.50%. Similarly, the ML integration intensity ratio at the knee joint ranges from 1.12 (angle: 18°) to 0.38 (angle: 90°), with a linear fitting factor of 98.24%. These ratios are almost within a 3–4 fold range and exhibit a uniform visual intensity ratio change with the increasing range of body movements. The high fitting factors also indicate the accuracy of human motion detection. Importantly, this bimodal intensity ratios sensing technology is not affected by the external environment, which will contribute to achieving a future mode of human‐machine collaboration, facilitating effective communication and cooperation between humans and machines to accomplish complex tasks.^[^ ^41^, ^42^ ^]^

## 3 Conclusion

In summary, a novel Tb^3+^/Mn^2+^ codoped Ca_9_Al(PO_4_)_7_ mechanoluminescent material with visualization, self‐referenced ML performance was successfully developed, and its ML performance under different strain were further studied after recombination with PDMS. CAP: 0.4Tb^3+^, 0.03Mn^2+^@PDMS composite elastomers can realize self‐referencing strain sensing techniques through the relationship between the integral intensity ratio of the two luminous peaks and strain during stretching. At the same time, when different shape variables are stretched, the ML color will change. From the visualization effect, the change of color has a higher spatial resolution than the change of intensity. In the process of ML, by adjusting the interaction between inorganic materials and organic materials friction electric potential, induced luminescence center force luminescence, further through the mechanical activation energy can be further transferred to the inner trap, resulting in the charging PersML performance, two different properties, can achieve attractive dynamic ML.

## 4 Experimental Section

### Synthesis of Inorganic Materials

Samples of Ca_9_Al(PO_4_)_7_ (CAP): 0.4Tb^3+^, yMn^2+^ (with y ranging from 0.03 to 0.4) were prepared using the high‐temperature solid‐phase method. The chemicals used in the synthesis included CaCO_3_ (99%), Al_2_O_3_ (99.99%), H_6_NO_4_P (99%), Tb_4_O_7_ (99.99%), and MnO_2_ (99.99%). The experimental procedure is as follows: First, the desired amounts of each chemical were accurately weighed using an electronic balance. The weighed chemicals were then placed in an agate mortar and mixed thoroughly with anhydrous ethanol. The mixture was ground in the mortar until the ethanol completely evaporated, resulting in a well‐mixed powder. Next, the powder mixture was transferred into a corundum crucible and sintered in an air atmosphere at 1200 °C for 8 h. After the sintering process, the sample was allowed to cool to room temperature and then further ground in a ceramic mortar to obtain a fine powder. The final powdered sample was used for subsequent measurements and analysis.

### Fabrication of ML Devices

First, 5 g of PDMS base resin (Sylgard 184, Dow Corning) and 0.4 g of curing agent were accurately weighed and transferred to a beaker for mixing. The mixture was then stirred using a magnetic stirrer for 10 min. Next, 2.5 g of the powder was weighed using an electronic balance and added to another beaker. The powder mixture was stirred using a magnetic mixer for an additional 10 min. Finally, the mixture was poured into a rectangular petri dish with dimensions of 100 mm (length), 100 mm (width), and 5 mm (height). The dish was left at room temperature for 5 min before being cured at 80 °C for 2 h. This process resulted in the fabrication of the ML film.

### Characterizations

The X‐ray diffraction (XRD) patterns were recorded by Germany Brucker D2 PHASER in the 2θ range of 10–80° by a step scan mode (step size: 0.02°; count time: 0.1 s per step) equipped with‐ Cu Kα radiation (λ = 1.5418Å) at 40 kV and 40 mA. The crystal structure was refined by the Rietveld method using the General Structure Analysis System (GSAS) program. Sample morphology was measured by using a scanning electron microscope (SEM) TESCAN VEGA 3 SEM (Tescan China, Ltd). Energy dispersive spectrum (EDS) and elements mapping (EM) were obtained by using Table electron microscopy (Ametek Materials Analysis Division). The photoluminescence (PL) and PL excitation (PLE) spectra were recorded with a Hitachi F‐7000 fluorescence spectrophotometer with a 150‐W Xe lamp as the excitation source. FLS980 Spectro‐flfluorometer equipped with a Xe lamp (made in UK Edinburgh industry) was used for testing the decay time at room temperature (RT) of the samples. The TL curves were measured with an FJ‐427A TL meter (Beijing Nuclear Instrument Factory, Beijing, China). The ML spectrum was captured by Ocean Insight (QE pro) when stretched by the Multi‐mode mechanoluminescence detection system (QKLN‐ML‐2). ML signals were collected in situ from a rotary friction testing machine (MS‐T3001). The exclusive photos were captured by the visible camera (Nikon D7500). The cathodoluminescence (CL) spectrum was detected on the modified Mp‐Micro‐S instrument attached to the SEM. The triboelectric properties were detected through the CSM friction testing machine (Tribometer 3, Switzerland) equipped with an electrostatic measuring probe (SK050, KEYENCE (Japan) Co., Ltd.). The distance from the friction interface to the electrostatic measuring probe is fixed at 10 cm, and the CSM friction machine adopts a rotating module with a rotation radius of 3 mm. The TD‐3000 diffractometer (Cu–Ka) is used as the X‐ray light source in all X‐ray (RL) tests. The emitted X‐ray wavelength is λ = 0.15405 nm.

### Friction Test

The mechanical behavior was analyzed using a friction testing machine (MS‐T3001), with ML signals collected in situ via a fiber spectrometer (QE Pro, Ocean Optics). For the friction experiments, various weights were applied to the friction pair to modify the load conditions. During the friction process, dynamic spectra were recorded using marine optical instruments, employing an integration time of 1 s.

### Strain Test

The strain experiment was conducted using a tensile testing machine equipped with a 100N pneumatic fixture. The tensile travel strain ranged from 0% to 100%, with a gauge length of 20 mm and a tensile speed of 16.6 mm s^−1^. All dynamic spectra were collected using the Ocean Optics QE‐Pro with an integration time of 300 ms.

The written informed consent was obtained from the volunteer, who is also a co‐author of this study. The volunteer has been fully informed of all experimental details, and measures have been taken to ensure their health and safety throughout the experiment.

### Patient Consent Statement

I declare that I am the patient whose information is included in the manuscript entitled “Trap Assisted Dynamic Mechanoluminescence Toward Self‐referencing and Visualized Strain Sensing”. I understand that the manuscript will be published and that my personal and medical information may be disclosed, and I hereby give my consent for the publication of this information. I have been informed of the purpose of the manuscript and the type of information that will be included. I am aware that my identification will be kept confidential and that my personal information will only be used for research purposes.

I understand that my participation in this research is voluntary, and I am free to withdraw my consent at any time. I have been provided with an opportunity to ask any questions regarding my participation, and all my questions have been answered. I further understand that my withdrawal of consent will not affect the quality or outcome of the research.

I understand that my consent is necessary for publication, and I consent to the publication of my personal and medical information in the manuscript.

## Conflict of Interest

The authors declare no conflict of interest.

## Author Contributions

T.W. performed data curation, wrote the original draft. P.Z. performed conceptualization. J.X. performed data curation. Z.G. performed software and validation. X.X. performed investigation. J.H. performed methodology. J.Z. performed methodology. X.X. performed conceptualization. L.Z. wrote and perform editing.

## Supporting information



Supporting Information

## Data Availability

The data that support the findings of this study are available from the corresponding author upon reasonable request.
