# Supplementary material for: Trap Assisted Dynamic Mechanoluminescence Toward Self‐Referencing and Visualized Strain Sensing
Source: Adv Sci (Weinh). 2024 Nov 27;12(3):2410673. doi: 10.1002/advs.202410673 (PMC11744709; doi:10.1002/advs.202410673)
Supplement: Supplementary file 1 — Supporting Information [file ADVS-12-2410673-s001.docx]

**Supporting Information**

**Trap Assisted Dynamic Mechanoluminescence Toward Self-referencing and Visualized Strain Sensing**

*Tianli Wang^a^, Pengfei Zhang^a^, Jianqiang Xiao^b^,Ziyi Guo^a^, Xiongwu Xie^a^, Jiahao Huang^a^, Jiaojiao Zheng^a^, Xuhui Xu^b,^*, Lei Zhao^a,^**

1. *School of Physics and Opto-Electronic Technology, Collaborative Innovation Center of Rare-Earth Optical Functional Materials and Devices Development, Baoji University of Arts and Sciences, Baoji, Shaanxi 721016, P. R. China.*
2. *College of Materials Science and Engineering, Key Laboratory of Advanced Materials of Yunnan Province, Kunming University of Science and Technology, Kunming, Yunnan 650093, China.*

** Corresponding author’s E-mail:* [*xuxuh07@126.com*](mailto:xuxuh07@126.com) *(Xuhui Xu),*

[*zhaoleibjwl@163.com*](mailto:zhaoleibjwl@163.com) *(Lei Zhao).*





**Figure S1.** XRD patterns of Ca_9_Al(PO_4_)_7_:*x*Tb^3+^ (*x* = 0.01-0.5) and the standard JCPDS card (No.48-1192);





**Figure S2.** XRD patterns of Ca_9_Al(PO_4_)_7_:*y*Mn^2+^ (*x* = 0.03-0.4) and the standard JCPDS card (No.48-1192);





**Figure S3.** XRD patterns of Ca_9_Al(PO_4_)_7_:0.4Tb^3+^,*y*Mn^2+^ (*x* = 0.03-0.4) and the standard JCPDS card (No.48-1192);





**Figure S4.** XRD Rietveld refinement of CAP:0.4Tb^3+^,0.03Mn^2+^;

**Table S1.** Final refined structural parameters for CAP;

| **Formula** | **CAP** |
| --- | --- |
| **Crystal system** | **trigonal** |
| **Space group** | **R3c (161)** |
| **Vol (Å^3^)** | **3386.344** |
| **Unit cell dimens (Å)** | **a = b = 10.270 Å c = 37.071 Å** |
| **Reliability factors** | **Rwp = 9.95 % Rp = 7.71 %** |
| **Program** | **GSAS** |
| **Profile range (deg.)** | **5 - 80** |


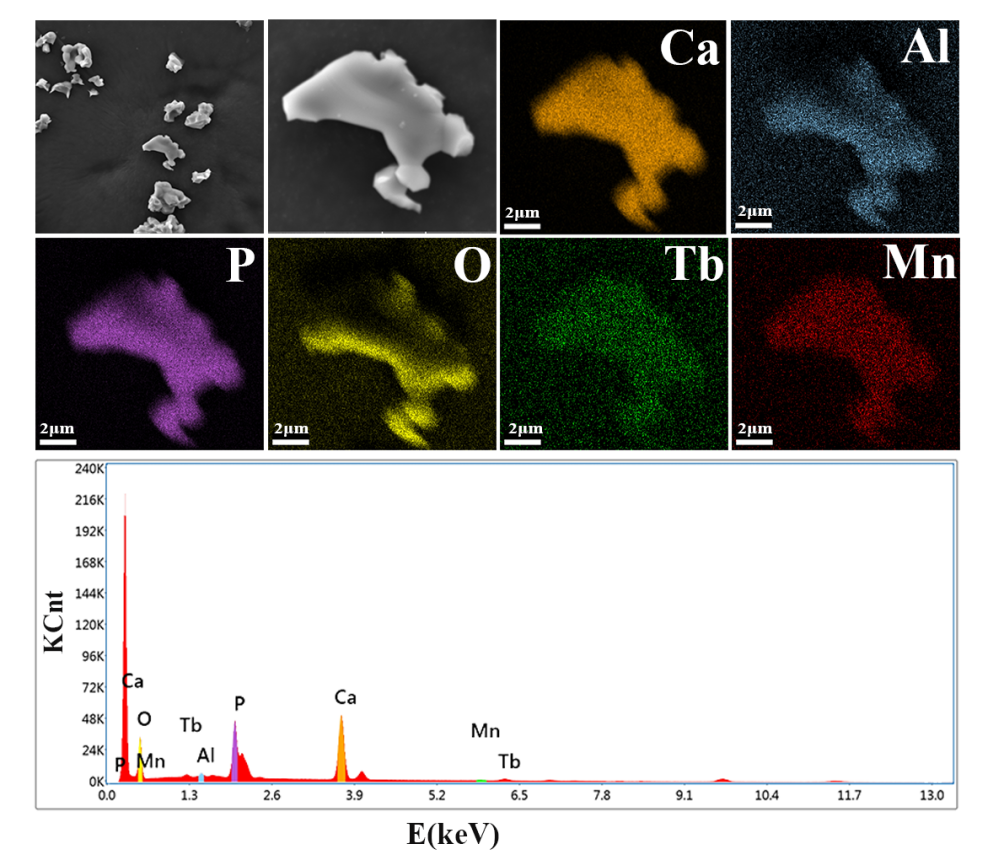


**Figure S5.** Elemental mapping of the representative particle of sample CAP:0.4Tb^3+^,0.03Mn^2+^ and EDS analysis of CAP:0.4Tb^3+^,0.03Mn^2+^;





**Figure S6.** PLE (λ_e_*_m_* = 548 nm) spectra of CAP: *x*Tb^3+^ phosphor, the inset is PLE emission intensity dependent on the concentration of Tb^3+^ ions;





**Figure S7.** PL (λ_e_*_x_* = 367 nm) spectra of CAP: *x*Tb^3+^ phosphor, the inset is PL emission intensity dependent on the concentration of Tb^3+^ ions;





**Figure S8.** PLE (λ_e_*_m_* = 648 nm) spectra of CAP: *y*Mn^2+^ phosphor, the inset is PLE emission intensity dependent on the concentration of Mn^2+^ ions;;





**Figure S9** PL (λ_e_*_x_* = 406 nm) spectra of CAP: *y*Mn^2+^ phosphor, the inset is PL emission intensity dependent on the concentration of Mn^2+^ ions;





**Figure S10.** PL (λ_e_*_x_* = 367 nm) spectrum of CAP: Tb^3+^ phosphor and PL (λ_e_*_x_* = 406 nm) spectrum of CAP: Mn^2+^ phosphor, the inset are PL pictures;





**Figure S11.** PLE (λ_e_*_x_* = 548 nm) spectrum of CAP:0.4Tb^3+^, *y*Mn^2+^ phosphor, the inset is PLE emission intensity dependent on the concentration of Mn^2+^ ions;;





**Figure S12.** PL (λ_e_*_x_* = 367 nm) spectrum of CAP:0.4Tb^3+^, *y*Mn^2+^ phosphor, the inset are PL pictures;





**Figure S13.** PL emission intensity dependent on the concentration of Mn^2+^ ions;


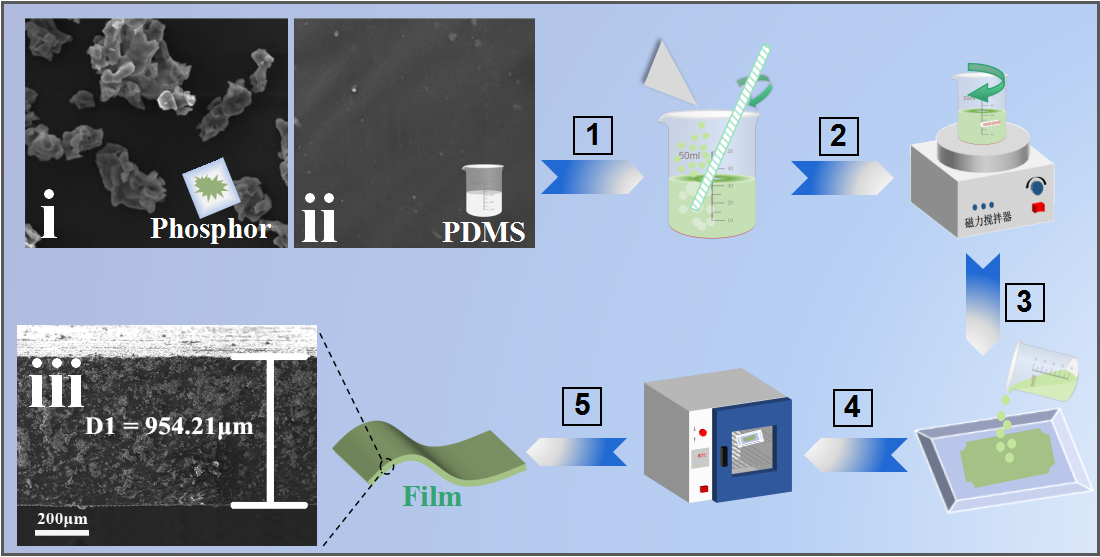


**Figure S14.** Schematic diagram of flexible film preparation;





**Figure S15.** XRD patterns of CAP:Tb^3+^， Mn^2+^@PDMS、PDMS、CAP:0.4Tb^3+^， 0.03Mn^2+^ and the standard JCPDS card (No.48-1192);


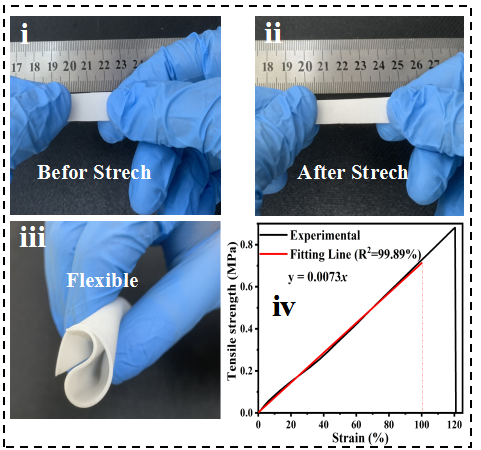


**Figure S16.** Composite film flexible display pictures, stress and strain curves;


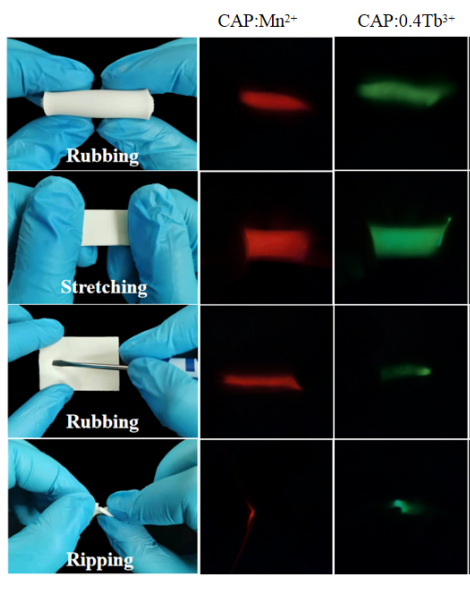


**Figure S17.** ML pictures of CAP:Tb^3+^@PDMS,CAP:Mn^2+^@PDMS under different force stimuli;





**Figure S18.** Self recovery of CAP:Tb^3+^@PDMS;





**Figure S19.** Self recovery of CAP:Mn^2+^@PDMS;


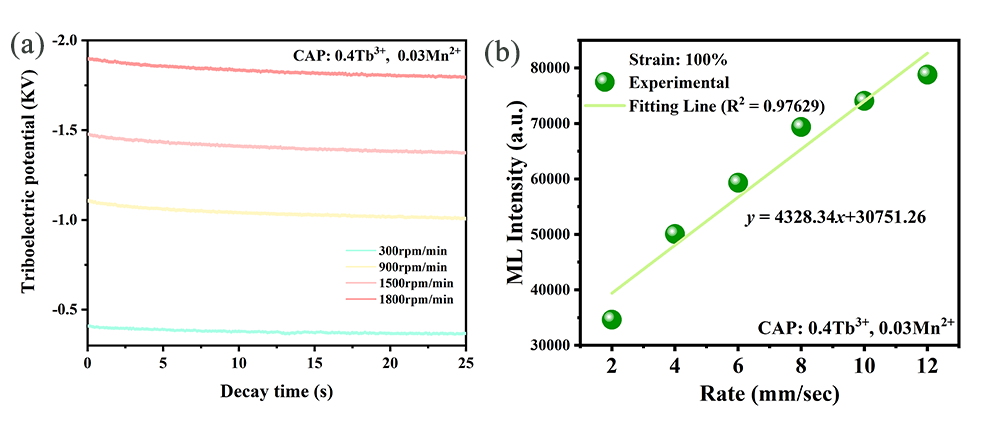


**Figure S20.** (a) Depicts the triboelectric potential of the CAP: 0.4Tb^3+^, 0.03Mn^2+^@PDMS composite device under different speeds; (b) Depicts the ML intensity of CAP: 0.4Tb^3+^, 0.03Mn^2+^@PDMS composite device under various strains rate;


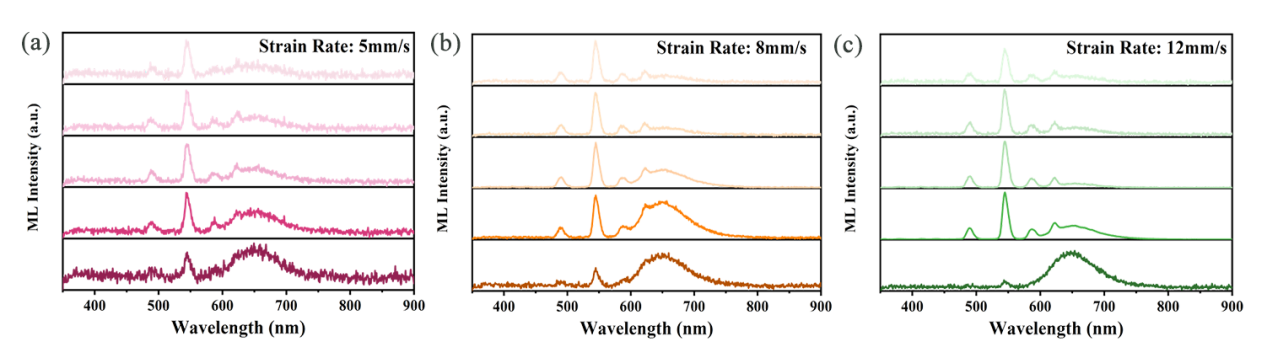


**Figure S21.** (a-c) Depicts the changes in ML spectra of the CAP: 0.4Tb^3+^, 0.03Mn^2+^@PDMS composite device at strain rates of 5 mm/s, 8 mm/s, and 12 mm/s, respectively；


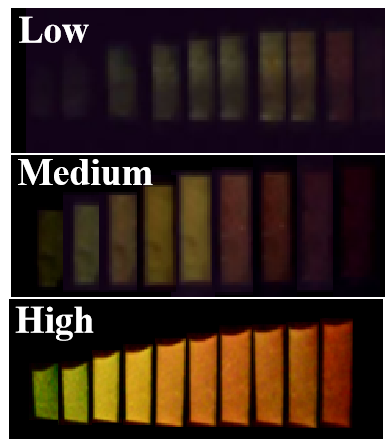


**Figure S22.** The photos of CAP: 0.4Tb^3+^, 0.03Mn^2+^@PDMS composite devices were taken at different strain rates;





**Figure S23.** CL spectra of CAP:Tb^3+^ at 70mA current and different driving voltages;





**Figure S24.** CL spectra of CAP:Mn^2+^ at 70mA current and different driving voltages;





**Figure S25** Persistent ML mappings of the CAP:Tb^3+^@PDMS composite elastomers;





**Figure S26.** Persistent ML mappings of the CAP:Mn^2+^@PDMS composite elastomers;


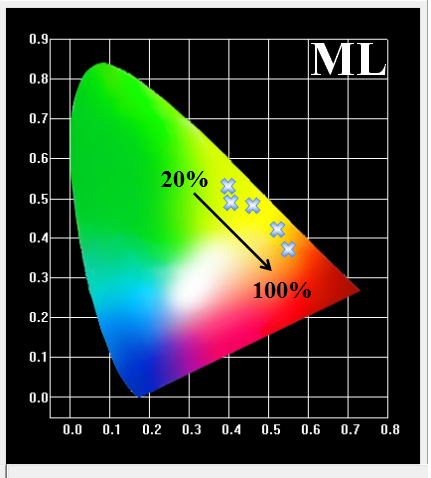


**Figure S27.** The chromaticity coordinates of ML;

**Table S2.** CIE of ML;

| **ML CIE(x,y)** | **x** | **y** |
| --- | --- | --- |
| **20%** | **0.3977** | **0.5299** |
| **40%** | **0.4038** | **0.4888** |
| **60%** | **0.4591** | **0.4777** |
| **80%** | **0.5251** | **0.4221** |
| **100%** | **0.5513** | **0.3715** |


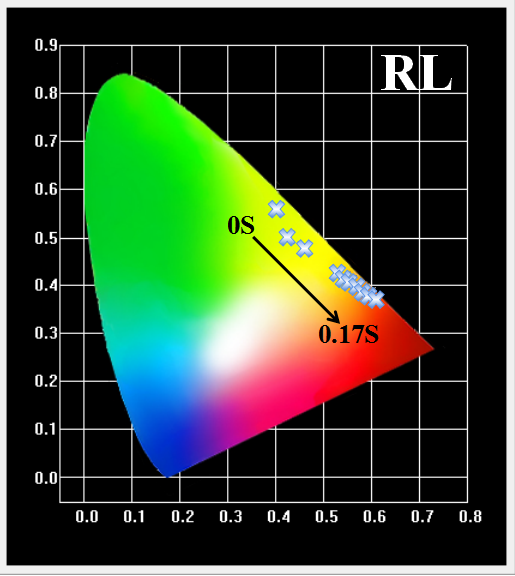


**Figure S28.** The chromaticity coordinates of RL;

**Table S3.** CIE of RL;

| **RL CIE(x,y)** | **x** | **y** |
| --- | --- | --- |
| **0.2s** | **0.4021** | **0.5612** |
| **0.4s** | **0.4238** | **0.4777** |
| **0.6s** | **0.4591** | **0.3094** |
| **0.8s** | **0.5299** | **0.4239** |
| **1.0s** | **0.5428** | **0.416** |
| **1.2s** | **0.5548** | **0.4084** |
| **1.4s** | **0.5709** | **0.398** |
| **1.6s** | **0.5829** | **0.3902** |
| **1.8s** | **0.594** | **0.3818** |
| **2.0S** | **0.6104** | **0.3696** |


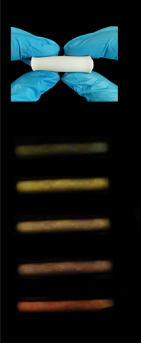


**Figure S29.** ML pictures of pinching;





**Figure S30.** CAP:0.4Tb^3+^,0.03Mn^2+^@ PDMS composite device ML spectra measured at different angles of elbow joint bending;





**Figure S31.** CAP:0.4Tb^3+^,0.03Mn^2+^@ PDMS composite device ML integral intensity ratio （I_Tb_/I_Mn_） of elbow joint bending at different angles；





**Figure S32.** CAP:0.4Tb^3+^,0.03Mn^2+^@ PDMS composite device ML spectra measured at different angles of knees joint bending;





**Figure S33.** CAP:0.4Tb^3+^,0.03Mn^2+^@ PDMS composite device ML integral intensity ratio （I_Tb_/I_Mn_） of knee joint bending at different angles；
